# “Young People Come to Youth Workers First”: A Mixed Methods Evaluation of the Role of Youth Workers in Youth Psychosis Detection

**DOI:** 10.1111/eip.70021

**Published:** 2025-02-26

**Authors:** Gabriele Gusciute, Ahmed Hassab Errasoul, Sean Naughton, Keith Gaynor, Niall Turner, Mary Clarke

**Affiliations:** ^1^ Dublin and East Treatment and Early Care Team (DETECT) Early Intervention in Psychosis Service Dublin Ireland; ^2^ School of Medicine Trinity College Dublin Dublin Ireland; ^3^ Department of Psychiatry, Faculty of Medicine King Saud University and King Khalid University Hospital Riyadh Kingdom of Saudi Arabia; ^4^ School of Medicine University College Dublin Dublin Ireland; ^5^ School of Psychology University College Dublin Dublin Ireland; ^6^ St John of God Community Services Dublin Ireland

**Keywords:** at‐risk mental state, early detection, early intervention, first episode psychosis, youth work

## Abstract

**Introduction:**

Reducing the duration of untreated psychosis (DUP) is a cornerstone of effective early intervention for psychosis (EIP) services. Delays in help seeking are a significant component of DUP, particularly among youth. Given their role as trusted adults in young people's lives, youth workers are uniquely positioned to play a role in early detection networks. However, their views regarding this remain largely unexamined.

**Aim:**

This study aimed to explore youth workers' perspectives on identifying and responding to psychosis in youth and the training needed to support them in this role.

**Methods:**

A sequential‐independent mixed methods design was employed. A brief questionnaire was given to youth workers (*n* = 86) who attended a 1‐day EIP training session, followed by semi‐structured interviews with a subset of participants (*n* = 10) 3 months later.

**Results:**

Most participants (98.8%) expressed confidence in recognising psychosis; however, stigma and challenging relationships with mental health services emerged as systemic barriers. Although most participants (98.8%) found the EIP training relevant to their role, they suggested it should form part of a broader mental health curriculum. There was a clear consensus among participants that such training is necessary and should be accessible, practical and actionable.

**Conclusion:**

Youth workers are uniquely positioned in identifying and supporting young people at risk of psychosis, and this study underscores their willingness to take on this role. To best support them, training efforts should focus on trans‐diagnostic approaches that enhance mental health literacy, address systemic barriers and promote multidisciplinary partnerships.

## 1 Introduction

The onset of psychosis can be a turning point in an individual's life. Its impact depends on several factors, one of the most modifiable being the duration of untreated psychosis (DUP) (Howes et al. 2021). Long DUP is associated with poorer outcomes including increased symptom severity, reduced likelihood of remission and impaired social and global functioning (Drake et al. 2020; Yu et al. 2023). In addition, it casts a long shadow, with adverse effects persisting decades after psychosis onset (O'Keeffe et al. 2022). This has focused attention on designing mental health services which are accessible to young people in the early stage of a psychotic illness, resulting in the early intervention for psychosis (EIP) model (Malla and McGorry 2019). Yet factors prior to initial assessment by specialised services continue to represent a barrier to engagement, with the interval between symptom onset and first mental health service contact thought to account for approximately one third of the DUP (Marino et al. 2020). This has prompted a shift in focus towards understanding the component delays that contribute to prolonged DUP, such as delays in help seeking (Malla et al. 2021).

The length of the pre‐service engagement DUP highlights the need to engage the broadest spectrum of professionals, including non‐healthcare community workers, to facilitate early help‐seeking behaviour (HSE National Clinical Programme 2019). Their input may be particularly important in youth mental health, as young people, despite recognising that they need professional help, often do not seek it (Dooley et al. 2019). This may partially account for the finding that a younger age of onset is associated with a longer DUP (Baeza et al. 2024). Against this backdrop, youth workers have emerged as key professionals in efforts to enhance early detection networks (Lynch et al. 2018). Situated at the intersection of child welfare, education and mental health, youth workers are uniquely positioned to recognise early warning signs of distress and to foster help‐seeking behaviours, leveraging their relational proximity and holistic understanding of young people's developmental needs (Ranahan and Thomas 2016). Despite this potential, pathways to care involving non‐clinical contacts have typically been associated with a longer DUP (Bhui et al. 2014), and interventions targeting community‐based agencies have so far proven largely ineffective (Oliver et al. 2018). For example, a community awareness programme offering direct referral pathways to EIP services failed to reduce the DUP or increase referral rates (Lloyd‐Evans et al. 2015). The authors cited a number of barriers such as stigma, fear, and negative past experiences with mental health services, which may deter community workers from making timely referrals.

There is a clear need to better understand how youth workers perceive their involvement in youth mental health generally, and in addressing psychosis specifically, so that the potential of their unique role can be harnessed to minimise treatment delays for young people. In addition, ambiguity remains regarding the scope of what mental health training for youth workers should entail, despite calls for its implementation (Department of Health 2017). This study aims to bridge this gap by exploring youth workers' views of their potential role in the early identification of and response to psychosis, including their perspective on the training necessary to effectively fulfil this role.

## 2 Materials and Methods

### 2.1 Participants

Participants were youth workers from various organisations across Ireland who attended a 1‐day EIP training course as part of a wider educational campaign to reduce DUP. Recruitment was facilitated by the National Youth Council of Ireland (NYCI), which advertised the course through online platforms and emails to affiliated organisations. The curriculum, detailed elsewhere (Hassab Errasoul et al. 2015), was designed specifically for youth workers and aimed to equip them with the skills to recognise psychosis and access appropriate treatment, with a strong emphasis on early intervention.

### 2.2 Study Design

To achieve both completeness and triangulation (Bryman 2006), the study employed a sequential‐independent mixed methods design (Schoonenboom and Johnson 2017). There were two sequential phases: a quantitative strand followed by a qualitative strand, allowing participants time to reflect on the training before interviews. Data collection in each strand was conducted independently, with neither contingent on the other's results. Equal weight was given to both strands, and integration occurred after data collection, allowing for examination of agreement, partial agreement, silence and dissonance according to the triangulation protocol (O'Cathain et al. 2010).

### 2.3 Quantitative Strand

A brief questionnaire, developed specifically for this study (Supporting Information S1), was administered immediately after the completion of the training course. Participants were asked to rate their agreement with statements evaluating the course and their perceived role in EIP using a 5‐point Likert scale (1 = strongly agree to 5 = strongly disagree).

### 2.4 Qualitative Strand

In‐depth, semi‐structured face‐to‐face interviews were conducted with a subset of workshop attendees. The interview questions were thematically aligned with the questionnaire, aiming to expand on participants' views. All participants were invited to take part. Recruitment continued until data quality signalled sufficient information power (Malterud et al. 2016), with the final number of participants determined by the relevance of the information gathered in answering the study's objectives.

### 2.5 Data Analysis

Survey data were analysed descriptively using Statistical Package for Social Sciences (SPSS) software, with one negatively worded item recoded to ensure that lower scores consistently reflected disagreement across all items. The interviews were transcribed verbatim and analysed using reflexive thematic analysis following Braun and Clarke's (2006) six‐phase framework: familiarisation, initial coding, theme development, refinement, naming, and reporting. A hybrid approach was employed, combining both deductive and inductive methods. While targeted interview questions and research aims guided the identification of anticipated thematic areas, codes were not predefined but instead emerged organically from the data. ATLAS.ti software facilitated qualitative data management.

### 2.6 Ethics

This study was approved by the research ethics committee of St John of God Hospitaller Ministries. Researchers provided detailed study information to potential participants and obtained written informed consent for research participation before data collection.

## 3 Results

### 3.1 Quantitative Strand

A total of 94 youth workers from various Irish youth organisations participated in the training, with 86 completing the survey. Among those who completed the training, participants had a mean age of 36.9 years (SD = 9.3), with the majority being women (*n* = 77, 82%) and Irish‐born (*n* = 78, 83%). The majority (*n* = 83, 88.3%) had attained at least a university‐level education or its equivalent. Most were employed as youth workers (*n* = 78, 83%) and had, on average, 8.1 years of experience in the field (SD = 6.9). While 65% (*n* = 61) had prior mental health training, primarily in suicide prevention, only 10.6% (*n* = 9) had received training specific to psychosis.

**FIGURE 1:**
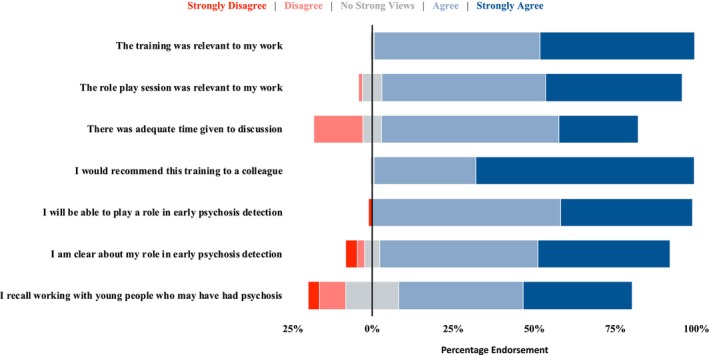
Distribution of participant responses to survey items indicating levels of agreement with statements concerning psychosis‐specific training and their role in detecting this in youth. Responses are displayed on a diverging scale, with agreement to the right of zero and disagreement to the left. Neutral responses (‘No Strong Views’) are evenly distributed across the midline.

There was a strong consensus among participants regarding all survey statements (Figure 1). The majority found the training relevant to their work (*n* = 85, 98.8%) and expressed a willingness to recommend it to colleagues (*n* = 85, 98.8%). Participants particularly valued the role‐play session (*n* = 79, 92.9%), although some suggested that additional time for discussion would have been beneficial (*n* = 13, 15.1%). Notably, 72.1% (*n* = 62) reported prior experience working with young people who they felt may have had psychosis. Overall, participants expressed greater confidence (*n* = 85, 98.8%) than clarity (*n* = 77, 89.5%) in their roles concerning the early detection of psychosis.

### 3.2 Qualitative Strand

Interviews were conducted with 10 youth workers engaged in services for individuals aged 8–25. Among the participants, six were employed in coordinator or managerial positions, while the remaining four held voluntary posts. While one participant's role was in supporting other youth workers, the remaining nine participants had roles that involved direct engagement with youth.

The thematic analysis identified two broad categories of themes. The first focuses on youth workers' perspectives regarding their role in EIP, with one theme describing the nature of this role, along with four themes representing facilitators and four outlining barriers to fulfilling it (Table 1). The second set of five themes explores youth workers' perspectives on their training needs and preferences for how this training should be delivered (Table 2). A summary of each theme, the topics they address, and relevant supporting quotes is provided in the tables below.

**TABLE 1:** Themes and quotes regarding the role of youth workers in EIP.

Theme	Brief description	Illustrative quotes
Topic: What should the role of youth workers be in EIP?		
Guiding the way: Youth workers as links to mental health care	Youth workers see themselves as supportive intermediaries who can bridge the gap between young people and mental health services, encourage help‐seeking and offer emotional support	“*They should play a part in promoting, let's say positive living, not to take on the role of professionals, but…to know where support is needed and to… support that young person in seeking support*” (P10, female youth work support officer)
Topic: What are the facilitators to this role?		
Beyond the call: Unwavering commitment to youth	Participants demonstrated a strong sense of personal responsibility and commitment, often going beyond their professional obligations to ensure young people received needed support	“*I am going to make it my business to do something about it. I don't know where I am going to get the funding to do it, but I have already brought a counsellor in that I bag packed to pay, working with two of our most vulnerable young people*” (P1, female youth worker in managerial role)
Being on the ground: Community connection and local insight	Youth workers underscored their deep‐rooted presence within the communities they serve and their intimate knowledge of the challenges faced by local families and the wider community	“*Youth Workers are a fountain of knowledge… they work on the ground with the young people in the young people's environment*” (P5, male youth project worker)
Trusted relationships: The heart of youth work	Participants envisioned the trusting relationships they cultivate with young people as their greatest asset allowing them to detect subtle behavioural changes and fostering a non‐judgemental space for sharing	“*The initial stage of diagnosis could be missed by the hospital or Mental Health Team, but it could be picked up in a Youth Work Centre…sometimes when young people go to doctors or hospitals it is a more formal setting and they don't feel as comfortable speaking the truth, whereas in the Youth Centre it is informal and they could have a better relationship built with the Youth Worker and they will say exactly how they are feeling*” (P2, female youth worker in managerial role)
Already on duty: Front‐line support	Youth workers described mental health as a central and unavoidable part of their evolving role as they are often the first point of contact for at‐risk youth dealing with poverty, family instability and substance abuse	“*In areas like this it is part and parcel of our job…If you are a Youth Worker and you're a good Youth Worker then you're noticing that Mental Health issues are out there….you can ignore it and say actually I am not trained to deal with it but then how you move that young person on or bring them on or help them reach their full potential*.” (P1, female youth worker in managerial role)
Topic: What are the barriers to this role?		
Caught in the cycle: Navigating the paradox of stigma	Pervasive stigma emerged not only as an obstacle for youth, but also the youth workers themselves who hesitated to use appropriate terminology and acknowledge mental health difficulties as an illness	“*The mental ‐ and I don't want to call it illness, because I want to call it wellness, I call it Mental Wellness*” (P1, female youth worker in managerial role)
Grounded in context: Mental illness through an environmental lens	Participants favoured a socio‐ecological view of mental illness which aligned with their preference for talking therapies and a belief that open dialogue could sufficiently address mental health challenges	“*I am not a Medical Practitioner, but a lot of them are put on drugs to help them overcome this and so when you are working with a 14 year old girl who is on Prozac and is having panic attacks, you have to question the system, I personally have to question the system… if somebody, somewhere along the line had just sat down and explained things to her*” (P1, female youth worker in managerial role)
Eroded trust: Frustration with mental health services	Participants voiced disillusionment with mental health services and felt undervalued by clinicians, citing tense interactions where their insights were overlooked	“*I did bring a chap to A&E to see a psychiatrist one night that had smoked crack cocaine… they were very reluctant to even talk to him and it was my persuasion that got them to actually keep him overnight….I realistically wanted the psychiatrist to give me a letter for my boss to say that he refused him care and of course he wouldn't give me a letter, so we fought for quite a while….I think I had a duty of care to this man because I know him and I think the psychiatrist had a duty of care because I am a professional and I was telling him*” (P7, female youth outreach worker)
Balancing act: Recognising boundaries and the limits of expertise	Participants indicated a need for clarity regarding their role expectations and discussed a preference for attempting to resolve issues themselves before seeking specialised support	“*If somebody came in depressed, we would try to find out what is causing the depression. If there is something going on at home or something going on in school, can we all fix it together, can they go with you and help sort that out. If it continues on and on and on and I am not able to fix it, then I would pass it onto somebody else…I would seek medical attention if they were very bad, but I would try to holistically fix it first*” (P1, female youth worker in managerial role)

**TABLE 2:** Themes and quotes regarding EIP training for youth workers.

Theme	Brief description	Illustrative quotes
Topic: Why is training in EIP important?		
The case for training: Knowledge breeds confidence	Participants reported that the training they received increased their awareness, preparedness and confidence in responding to psychosis and called for formalised and compulsory mental health education for youth workers	“*Before the course I was kind of thinking if it occurred I thought I would be able to deal with it and now I know I would be able to handle it and I am very confident in how I could approach the situation and it's like a relief that if the time comes and I do have to encounter that experience that I would be able to do it to the best of my ability with good knowledge on it*” (P8, female part‐time youth work volunteer)
Topic: Who should provide training?		
Training providers: A blend of expertise and practical wisdom	A collaboration between mental health professionals and experienced youth workers was seen as the most effective way to deliver credible, relevant and actionable training	“*You need to see where a Youth Worker is coming from, so it is not just a clinical training, that it is very much a Youth Workers training and it needs to be a blend of the two…I think the training that we did in February was great to have a Mental Health Professional…to have somebody that could answer your questions…I think it should be developed jointly and delivered jointly*” (P9, female youth health worker)
Topic: Who should receive training?		
Training receivers: Universal access, targeted expertise	Participants called for universal access to basic mental health training, with advanced training reserved for those working with at‐risk youth or a dedicated youth worker within each service	“*It is like the idea of the suicide, where everybody was thinking an elite only should get the suicide training, whereas now ASSIST is completely democratized and everybody is having access to ASSIST, which has been fantastic even for Youth Workers to build their confidence in dealing with suicide…It is about making it available to everybody but in terms of going into details, maybe more Youth Workers that have more of a chance to actually apply it*” (P6, male youth health programme co‐ordinator)
Topic: What should be the content of the training?		
Training curriculum: Building blocks in action	Participants preferred incorporating psychosis training into a broader, foundational framework that covered commonly encountered issues such as anxiety and depression and focused on identifying symptoms, making referrals, and managing group dynamics when mental health issues arise	“*I wouldn't put psychosis out there on its own; I would put it under the umbrella of Mental Health issues…Like if I have the flu or pneumonia or something I don't separate the flu as one bit and all the other bits are there…It shouldn't take that much of a space… I found it was too deep, too heavy for me … I would have needed to be brought along slower*” (P1, female youth worker in managerial role)
Topic: How should the training be delivered?		
Training delivery: A hands‐on approach for real‐world readiness	Participants emphasised interactive and practical training methods (e.g., role plays) in best preparing them for the on‐the ground‐challenges they may face. There was a preference for in‐person training as it offered an opportunity to connect with peers, share experiences and offer mutual support	“*It would have to be practical because that is what the work is here, practical and hands on…people that work in a situation like this, they want to know what if someone came in and they were just acting this way, what do I do?*” (P3, female youth worker in managerial role)

## 4 Triangulation

### 4.1 Agreement

The quantitative and qualitative strands collectively highlighted participants' confidence in their ability to identify and manage psychosis, despite acknowledging a need for further clarity regarding the limits of this role. Regarding the training, a preference for practical hands‐on methods, such as role play, was widely supported by the quantitative data and also surfaced in the qualitative findings as crucial for preparing youth workers for real‐life scenarios. Both strands of data also highlighted the importance of allowing sufficient time for questions and discussion, which allowed participants to seek clarification and integrate their newly acquired knowledge into their professional practice.

### 4.2 Partial Agreement

Overall, the study revealed a strong endorsement for EIP training, with most participants affirming its relevance in the survey responses. However, the interviews provided a more nuanced perspective, suggesting that while participants recognised the training's critical role in addressing knowledge gaps, they felt psychosis should not be taught in a vacuum, but rather contextualised within broader and universally available mental health training.

### 4.3 Silence

The qualitative data highlighted themes not directly addressed by the survey. Participants emphasised that youth mental health is already a fundamental part of their role, reinforced by the trusting, voluntary relationships they build with young people and their deep understanding of the challenges these youth face. However, despite their willingness to support young people as they navigate mental health services, stigma among the participants emerged as a pervasive challenge. It was subtle and largely unacknowledged by the youth workers, who, despite framing mental illness as a health issue, hesitated to use appropriate terminology and fully recognise it as an illness. In addition, the interviews expanded on participants' views regarding the format of EIP training, underscoring the value of co‐facilitation by both mental health professionals and experienced youth workers, as well as a strong preference for in‐person training, which was perceived as crucial for fostering networking opportunities.

### 4.4 Dissonance

Quantitively, participants expressed a high degree of confidence in their ability to recognise and address psychosis in youth. However, the qualitative data underscored a disconnect between their sense of preparedness post‐training and the broader systemic issues they encounter, including a lack of shared understanding, support and recognition of their role from mental health services. Youth workers articulated their role in opposition to that of clinicians, particularly in terms of emotional involvement and commitment to youth. Their perspectives on managing mental illness also diverged from those of mental health teams, especially concerning the use of pharmacotherapy and the conditions warranting inpatient admission.

## 5 Discussion

Youth workers in this study viewed their involvement in youth mental health not as a diversion, but rather a natural and necessary evolution of their role. While some have described youth workers best suited to act as gatekeepers to mental health services (Rickwood and Mazzer 2012), others contend that this leaves them ‘positioned merely at the doorway, facilitators to other service providers, waving young people through but not accompanying them to the other side’ (Ranahan and Pellisier 2015, 231). Instead, proponents of the latter view have advocated for youth workers to be fully integrated within collaborative mental health teams, a model already implemented elsewhere (Rickwood et al. 2019). Participants in this study envisioned their role as falling somewhere in the middle of these perspectives, seeing themselves as ‘in‐betweens’, supportive bridges between young people, their families and specialised services. They emphasised that their greatest strength lies in their position as trusted adults for vulnerable young people often disconnected from formal support systems. However, this advantage may be compromised should their role become more formally integrated into mental health services, as the trust youth workers build could be strained—particularly when they must prioritise a young person's safety by involving others without the young person's consent (Ranahan 2013). This risk is heightened in cases of psychosis, where young people may lose insight and disengage from support entirely (Kim et al. 2019). Nevertheless, youth workers are already an obligate part of the intervention pathway given that they cannot control or anticipate the mental health challenges that young people under their care will present. Therefore, the discourse must now shift from questioning their role to exploring how best to support and equip them for success. This study highlights several potential pitfalls that need to be addressed to ensure their involvement is beneficial and does not undermine the very relationships that make them effective.

A key consideration in preparing youth workers to address the mental health challenges of young people lies in determining the most effective approach to increasing their mental health literacy. Participants in this study recognised the relevance of EIP training; however, they noted that a focus on psychosis is premature without a solid grounding in general mental health principles. As their role is not to diagnose but rather to identify when a young person is unwell and in need of specialised support, perhaps training should focus less on diagnostic distinctions and more on recognising early signs of distress and functional decline, indicators that warrant closer monitoring or referral to secondary services (Fusar‐Poli et al. 2014). This aligns with evidence suggesting that the period preceding a psychotic illness is often characterised by non‐specific indicators of risk such as increased help seeking, suicidal behaviour, and social isolation (Sullivan et al. 2018), offering a critical window for early intervention before the die is cast. Although identifying those at risk can be challenging even for health professionals (Strelchuk et al. 2021), youth workers are uniquely positioned to notice subtle yet persistent changes in social engagement and daily functioning due to their consistent presence and deep knowledge of young people's lives. In addition, condition‐specific workshops may inadvertently reinforce participants' belief that illnesses such as depression and anxiety are distinctly separate from psychosis; whereas, in practice, the boundaries between these conditions often blur. This is particularly true during the prodromal phase, where, for example, differentiating between depression and delusional mood can be challenging (Fusar‐Poli et al. 2012). Thus, while psychosis‐specific training yielded positive outcomes, a more effective strategy may involve a comprehensive trans‐diagnostic curriculum that encourages a focus on identifying risk rather than on a diagnosis, akin to Mental Health First Aid, which has shown promise in this regard (Ng et al. 2021).

This study revealed that didactic training alone may be insufficient in sustaining youth workers' role in EIP without simultaneously addressing existing barriers. For example, stigma is not only pervasive, but can undermine the effectiveness of community mental health literacy initiatives (Lloyd‐Evans et al. 2015). In this study, youth workers expressed a reluctance to use appropriate psychiatric labels. While this aligns with their socio‐ecological perspective on mental illness, it also introduces several challenges. Developing comfort with accurate terminology not only facilitates effective communication within multidisciplinary teams but also plays a crucial role in reframing mental illness as a medical condition rather than a personal failing (Wright et al. 2011). This is particularly important given that youth workers expressed a preference for psychotherapy and viewed medication as indicative of a failure to intervene early. This perspective is echoed in the youth work literature, which reflects a degree of scepticism towards what is perceived as an overly medicalised approach to youth mental health, with youth work positioned as a counter‐narrative rather than a cooperative ally (Elsen and Ord 2023). This view is likely shaped by the significant experience youth workers have with young people facing age‐appropriate difficulties, where counselling may be more appropriate. However, addressing serious mental illnesses such as psychosis demands a broader, more comprehensive treatment approach (Fusar‐Poli et al. 2017). Therefore, educational initiatives for youth workers should adopt a twofold strategy. First, they must tackle the implicit stigma that some workers may hold. Curricula that incorporate service user testimonies (Thornicroft et al. 2016) and experiential arts interventions (Gaiha et al. 2021) have shown promise in this regard and align with the relational, participatory ethos central to youth work practice (Ranahan and Pellisier 2015). Second, training should offer a thorough overview of biopsychosocial treatment planning, emphasising the multimodal nature of EIP interventions and demonstrating that while psychotherapy is a vital element, it is only one part of a more holistic, integrated approach to managing psychosis.

Another significant barrier that repeatedly surfaced in the interviews is the difficulty youth workers face in interfacing with mental health services. Although EIP training can enhance their understanding of psychosis, it may ultimately fail in the goal of promoting help‐seeking behaviours. Without a belief in the effectiveness of mental health services, this knowledge may fall short in practice (Lloyd‐Evans et al. 2015). The interviews highlighted an adversarial ‘us versus them’ mentality which may reinforce a hierarchical dynamic, implicitly positioning clinicians as gatekeepers of legitimacy, thereby stifling youth workers' professional autonomy and hindering the development of genuine, collaborative relationships (Ranahan and Thomas 2016). Such tensions were evident in accounts of youth workers feeling dismissed, such as when inpatient care was denied despite their concerns. Frustrations understandably arise when the rationale for such decisions, such as admission not being appropriate or beneficial, is poorly communicated, and may feed into a broader belief that mental health professionals are insufficiently invested in helping young people. Addressing these tensions requires targeted training that positions the roles of youth workers and mental health professionals as complementary and mutually reinforcing, rather than oppositional. Efforts must be made on both sides: youth workers need to gain a deeper understanding of the structure and decision‐making processes within mental health services, while clinicians must acknowledge the role of youth workers extends beyond mere custodial duties.

Despite their negative experiences, youth workers expressed a desire for more effective communication and collaboration with mental health teams. While there may be a rationale for a more direct link with EIP services, allowing at‐risk youths to be referred immediately to EIP services, this approach would have to be considered carefully given the likelihood of a high proportion of ‘non‐case’ referrals in the context of low‐risk enrichment in community samples and the non‐clinical background of many youth workers (Fusar‐Poli et al. 2019). Where this has been trialled, the strategy has been ineffective, with prior negative experiences with mental health professionals being a barrier to the referral processes (Lloyd‐Evans et al. 2015). This notwithstanding, it is important that youth workers feel supported when concerns about a young person arise so that they are not left feeling isolated and solely responsible for ‘holding’ a person in distress (Ní Charraighe and Reynolds 2024). One approach could involve assigning a dedicated liaison from the local mental health team to each youth service, offering guidance on concerning cases and advising on appropriate next steps. An alternative strategy may involve organisational policies that encourage direct communication between youth workers and clinicians (Ranahan and Thomas 2016), such as a recurring forum allowing for regular feedback between the two disciplines.

### 5.1 Strengths and Limitations

This study was conducted within a specific national context. The interface between youth workers and mental health services has varied configurations across different jurisdictions, from models where youth workers operate independently of mental health services to those where they are integrated within multidisciplinary teams, such as Australia's Headspace initiative (Rickwood et al. 2019).This should be considered in terms of its generalizability. However, these findings may also have broader international relevance given that communication between social care and healthcare professionals is a consistent feature of any interface. Thus, this study contributes to the broader, timely, and necessary discourse on expanding mental health education and responsibilities to non‐clinically trained community workers, highlighting both the challenges and opportunities this approach presents.

The strength of this study lies in its mixed methods design, which not only enabled the corroboration of findings across both strands but also uncovered nuances in youth workers' perspectives. While the views expressed may have been influenced by participants' interest in mental health and thus not fully represent the voices of other youth workers who have diverse backgrounds and priorities, their reflections represent an important contribution to service design and the research agenda. The independent structure of the study meant that interview sampling was not guided by survey responses. While this can ensure a broad range of viewpoints, it is unlikely to have influenced the findings, as participants generally showed strong agreement on the survey items. Lastly, participants in the qualitative phase reported limited exposure to psychosis, which reduced opportunities to apply the training before the interviews. Nevertheless, their insights remain valuable, as many youth workers likely have minimal experience with psychotic illness.

## 6 Conclusion

Participant feedback in this study highlighted both the need for psychosis training and the importance of refining its focus. Future iterations could emphasise practical, scenario‐based learning to equip youth workers with the skills to identify trans‐diagnostic, early indicators of mental illness, determine thresholds for secondary care referral, and address barriers that may prevent community members from encouraging help‐seeking in youth. It would be instructive to re‐evaluate the training following these refinements.

Finally, while this study highlights the enthusiasm of youth workers in supporting the mental health needs of young people, enthusiasm alone is not enough. None of the participants in this study had access to EIP services in their communities. Without expanding these services, the goals of EIP—and by extension, this study—risk remaining aspirational rather than attainable.

## Conflicts of Interest

The authors declare no conflicts of interest.

## Supporting information


**Supporting Information S1.** Brief Questionnaire.

## Data Availability

The data supporting the findings of this study are available upon reasonable request from the corresponding author. Data sharing is subject to institutional and ethical approvals.
