# Supplementary material for: “Young People Come to Youth Workers First”: A Mixed Methods Evaluation of the Role of Youth Workers in Youth Psychosis Detection
Source: Early Interv Psychiatry. 2025 Feb 26;19(3):e70021. doi: 10.1111/eip.70021 (PMC11862984; doi:10.1111/eip.70021)
Supplement: Supplementary file 1 — Supporting Information S1. Brief Questionnaire. [file EIP-19-0-s001.docx]

**Supplementary Material 1**

Brief Questionnaire

|  | Strongly Disagree | Disagree | No Strong Views | Agree | Strongly Agree |
| --- | --- | --- | --- | --- | --- |
| I have found the training relevant to my work |  |  |  |  |  |
| The session on role play was relevant to my work |  |  |  |  |  |
| Time given to discussion was adequate |  |  |  |  |  |
| I will recommend this training to a colleague |  |  |  |  |  |
| I think I will be able to play a role in early detection of psychosis |  |  |  |  |  |
| I feel confused about my role in early detection of psychosis |  |  |  |  |  |
| I can remember young people I have dealt with in the past who may |  |  |  |  |  |
